# A validation of ground ambulance pre-hospital times modeled using geographic information systems

**DOI:** 10.1186/1476-072X-11-42

**Published:** 2012-10-03

**Authors:** Alka B Patel, Nigel M Waters, Ian E Blanchard, Christopher J Doig, William A Ghali

**Affiliations:** 1 Department of Community Health Sciences, University of Calgary, Calgary, Alberta, Canada; 2 Department of Geography and Director, GIS Center of Excellence, George Mason University, Fairfax, Virginia, USA; 3 Alberta Health Services, Emergency Medical Services, Calgary, Alberta, Canada; 4 Department of Medicine, University of Calgary, Calgary, Alberta, Canada; 5 Institute of Public Health, University of Calgary, Calgary, Alberta, Canada

**Keywords:** Pre-hospital time, Geographic Information Systems, Validation, Emergency medical services

## Abstract

**Background:**

Evaluating geographic access to health services often requires determining the patient travel time to a specified service. For urgent care, many research studies have modeled patient pre-hospital time by ground emergency medical services (EMS) using geographic information systems (GIS). The purpose of this study was to determine if the modeling assumptions proposed through prior United States (US) studies are valid in a non-US context, and to use the resulting information to provide revised recommendations for modeling travel time using GIS in the absence of actual EMS trip data.

**Methods:**

The study sample contained all emergency adult patient trips within the Calgary area for 2006. Each record included four components of pre-hospital time (activation, response, on-scene and transport interval). The actual activation and on-scene intervals were compared with those used in published models. The transport interval was calculated within GIS using the Network Analyst extension of Esri ArcGIS 10.0 and the response interval was derived using previously established methods. These GIS derived transport and response intervals were compared with the actual times using descriptive methods. We used the information acquired through the analysis of the EMS trip data to create an updated model that could be used to estimate travel time in the absence of actual EMS trip records.

**Results:**

There were 29,765 complete EMS records for scene locations inside the city and 529 outside. The actual median on-scene intervals were longer than the average previously reported by 7–8 minutes. Actual EMS pre-hospital times across our study area were significantly higher than the estimated times modeled using GIS and the original travel time assumptions. Our updated model, although still underestimating the total pre-hospital time, more accurately represents the true pre-hospital time in our study area.

**Conclusions:**

The widespread use of generalized EMS pre-hospital time assumptions based on US data may not be appropriate in a non-US context. The preference for researchers should be to use actual EMS trip records from the proposed research study area. In the absence of EMS trip data researchers should determine which modeling assumptions more accurately reflect the EMS protocols across their study area.

## Background

Evaluating geographic access to health services often requires determining the patient travel time to a specified facility. For urgent travel by ground, geographic information systems (GIS) are gaining favor as a tool to model the pre-hospital time of Emergency Medical Services (EMS). Since patient EMS records can be difficult to collect at a national level, GIS allows spatial access to be modeled over large areas and multiple jurisdictions using readily available data. This method commonly uses digital road network data within GIS to model the transportation times from patient locations to hospitals over large geographic areas. In order to determine geographic access, studies often focus on using the GIS modeled time component from scene to arrival at hospital for large areas in the absence of actual trip data [1,2]. Although determining the transport time from scene to hospital is sufficient in some cases, in others there is a need to determine access in terms of total pre-hospital time.

There are multiple time intervals that are considered to be part of the total pre-hospital time [3]. Many studies have adapted a definition that considers the overall pre-hospital time as being comprised of four unique time intervals. These are the activation, response, on-scene and transport intervals [4]. The activation interval is the time from the emergency call to ambulance dispatch. The response interval is the time from ambulance dispatch to the ambulance arrival at the scene. The on-scene interval is the time from ambulance arrival at the scene to the time when the ambulance departs the scene for hospital. Finally, the transport interval is the time from ambulance departure from the scene to arrival at the hospital. These four time intervals combine to give the total pre-hospital time of a patient from the emergency call to hospital door. A meta-analysis has provided summary measures for these ambulance pre-hospital time intervals in the United States [4].

In recent years there have been multiple studies that have used the research conducted by Carr *et al.*[4] and Branas *et al.*[5,6] as the foundation to model national access to services by ground ambulance across the United States (US). While some of these studies have not specifically used modeled time along a road network calculated using GIS [7-9], recent studies have adapted their methods to include the use of GIS to calculate travel time along the road network [10,11]. Recently, a Canadian study has also used these pre-hospital time assumptions with GIS measured transport intervals to evaluate population access to Percutaneous Coronary Intervention facilities [12]. Although these models have been used to study access in both the US and Canada, their validity compared to actual EMS data has not yet been determined. Using a unique data set of EMS trip records for a large Canadian city, our study has two objectives: 1) Determine if the modeling assumptions proposed through prior studies are valid in a Canadian context and 2) use the resulting information to provide revised recommendations and assumptions for modeling travel time using GIS in the absence of actual EMS trip data.

## Methods

### Study setting

This study was approved by the Conjoint Health Research Ethics Board at the University of Calgary and was conducted within and surrounding a large Canadian city, Calgary. At the time of the study, the Calgary Health Region (CHR) administered all publicly funded hospital care, including emergency services in three tertiary care adult hospitals, to the residents of the cities of Calgary and Airdrie and approximately 20 nearby small towns, villages, and hamlets (population 1 million) in the Province of Alberta, Canada. The City of Calgary Emergency Medical Services was the sole provider of ambulance services to the City of Calgary and to the surrounding regions, which include the Town of Chestermere, the Tsuu T’ina Nation and sections of the Municipal District of Rockyview.

This EMS system had approximately 44 response units, all of which were Advanced Life Support equipped and staffed. In 2006, this service recorded 107,562 EMS unit responses [13]. Based on information provided by the caller, and interpreted by a registered emergency medical dispatcher using the Medical Priority Dispatch System (MPDS), emergency situations were identified and given the designation of Alpha, Bravo, Charlie, Delta, or Echo level events. The MPDS rates the emergency from least serious (Alpha) to most critical (Echo) [14]. The dispatch of EMS units in this jurisdiction using the MPDS is consistent with industry-accepted quality standards. In the jurisdiction for this study, Alpha level calls receive an ambulance response without lights and sirens (non-emergency). Bravo, Charlie, Delta, and Echo calls receive an ambulance response using lights and sirens, and Delta and Echo level calls also receive a response by the fire department, who provide Basic Life Support with defibrillation medical first-response and scene assistance [13].

### Study sample

The study sample contained 31,385 patient trips (for adults 18 years of age or older) within the Calgary area between January 1 and December 31, 2006. A full year of data allowed for the daily and seasonal fluctuations in EMS trips to be accounted for over the entire study period. All calls contained in this sample were either a Bravo, Charlie, Delta or Echo level call, which means that the caller provided key information that led the dispatcher to conclude that the call was for a time-dependent emergency [14]. This ensured that the travel times considered were for ‘emergency’ response by EMS. Functionally two ambulances dispatched from the same location to the same location would arrive at the same time even if one was a Delta and the other Bravo, as the response is the same. The ‘lights and sirens response’ is the only difference in the priority of dispatch, but if two calls are made at the same time, the closest ambulance will be dispatched to the higher level call.

### Study variables

Each record contained a patient location at the time of call. The location was recorded as an address, an intersection or a common place name. Each patient location was converted to an x/y coordinate by CHR analytic support staff within the administrative data group to ensure precise representation of the patient event location. Each record also contained the hospital address to which patients were transported. This data was prepared for a prior study focused on the associations between emergency response time and mortality in an urban setting [13]. Records that did not include patient event or transport location information were excluded from this study. Records for scene locations within the city boundary were categorized as urban, while those for scene locations outside of the city were categorized as rural. In previous studies, categorizations of urban, suburban and rural have been made based on tertiles of population density [5,10]. Our method of dividing the scene locations by inside and outside the city limits yielded comparable categorizations to the methods used from these previous studies.

Each record also contained a time stamp that identified the start of the activation interval, response interval, on-scene interval and transport interval for each patient trip. The activation interval was defined using the time stamps from when the time the emergency call was received to the time the ambulance was en-route to an event. The response interval was defined using the time stamps from the time the vehicle was en-route to the time it arrived on scene. The on-scene interval was defined using the time stamps from the time the ambulance arrived on scene to the time it left the scene. Finally, the transport interval was defined using time stamps from the time the vehicle left the scene to the time it arrived at the hospital. The time stamp corresponding to when the emergency call was received was automatically generated by the EMS computer aided dispatch (CAD) system upon receipt of the call. All other timestamps were automatically recorded by the CAD system when the responding paramedic pressed the appropriate button on a mobile data terminal in the vehicle. Records with any missing time stamps were excluded from this study.

### GIS data and travel time analysis

The transport interval is the time interval that can be modeled using GIS. This time component is the travel time along the road network from patient scene location to the hospital. Travel time along a road network requires data representing origins of travel, destinations and the linear features along which travel occurs. The origins in this study were the recorded scene location of patients; the destinations were the geocoded hospital emergency department locations. The road network we used was the CanMap® RouteLogistics file (DMTI Spatial, Markham, Ontario). This file can be used for shortest route analyses of both time and distance. In addition to containing detailed street names and address locations along each segment of road, fields are included for the length and speed limit along each segment of road.

This GIS derived transport time from scene to hospital was subsequently used to model the response interval (time from dispatch to patient scene). In many study areas, the location of the ambulance at the time of dispatch is unknown. For example, the EMS database that we used does not consistently include the location of the ambulance at the time of dispatch. In past studies, in the absence of information on ambulance locations at time of dispatch, empirically derived constants based on the literature were used to account for the time for an ambulance to reach a patient [5,10,12]. Using actual ambulance data, these empirical constants were derived by determining the relationship between the response and transport intervals. GIS derived travel times from scene to hospital were multiplied by 1.6 to obtain overall travel times in urban areas and 1.4 in rural areas [5,6]. This means that the response interval was modeled to be 60% and 40% of the transport interval in urban and rural areas, respectively.

### Objective 1: Validation of EMS modeling assumptions

The overall time from emergency call to hospital requires a representation of the various time factors that constitute pre-hospital time. Below is a description of how comparisons between the actual EMS time factors captured in our database were made with the time factors as described in previous literature or derived using GIS. All statistical analyses were conducted using Stata 10.0 [15].

#### Activation interval and On-scene interval

Descriptive analysis was conducted with the entire study sample to determine the actual median activation and on-scene intervals. The sample was divided into within city and outside city in order to understand urban/rural differences in activation and on-scene times. A comparison with the average activation and on-scene interval from past studies was made using boxplots.

#### Response interval

The actual response interval is contained within the EMS data. Descriptive analysis was conducted with the entire study sample to determine the median time to patient. The sample was divided into within city and outside city in order to understand urban/rural differences in the response interval. A comparison was made using scatterplots between the actual response interval and the times derived from the GIS model using empirical multipliers.

#### Transport interval

We used the Network Analyst extension of Esri ArcGIS 10.0 [16] to estimate travel time by ground from the scene location to the hospital destination for each trip in the EMS database. Travel cost matrices were used to determine the shortest route in minutes from each patient scene location (geocoded from their x/y coordinates) to one of the three tertiary care centers as described in the database.

The actual transport time from scene to hospital is contained within the EMS database. Descriptive analysis was conducted with the entire study sample to determine the median transport time. The sample was divided into within city and outside of city in order to understand urban/rural differences in the transport time. Using scatterplots, a comparison was made between the actual transport intervals and those derived from the GIS model.

#### Total pre-hospital time

The overall pre-hospital time is derived as a sum of the four above-mentioned time components and can be described as follows:

1) Activation interval + Response interval + On-scene interval + Transport interval

The EMS database contains the actual total pre-hospital time from emergency call to hospital. The model for overall time is as follows where ‘GIS transport’ is the time modeled from scene to hospital using GIS and defined assumptions are from published literature [4,6]:

2a) Urban: 1.4 minutes + (0.6*GIS transport) + 13.5 minutes + GIS transport

2b) Rural: 2.9 minutes + (0.4*GIS transport) + 15.1 minutes + GIS transport

The actual and modeled overall pre-hospital times were evaluated for statistically significant differences using the Wilcoxon signed-rank test. Using scatterplots, a comparison was made between the actual times from emergency call to hospital and these times derived using GIS and published literature. The differences between these two times were mapped to gain an understanding of the underestimation and overestimation of the model over the study area. All GIS analyses and map production were conducted using Esri ArcGIS 10.0 [16].

### Objective 2: Revising EMS modeling assumptions for a Canadian setting

The second objective of this study was to create a model that more accurately reflected total EMS pre-hospital time in a Canadian context. We used the information acquired through the analysis of the EMS trip data as outlined above, to develop a model that could be used to estimate travel time in the absence of actual EMS trip records. The creation of such models has been useful for estimating the access of a population across a large study area [5,10,12].

The actual EMS median dispatch time and time at patient scene were used to represent the dispatch time and time at scene respectively. The transport time from scene to hospital was modeled using GIS network analysis from patient scene location to hospital using the shortest time algorithm. The response interval was subsequently modeled using linear regression with no y-intercept to determine the relationship between the GIS modeled transport interval and the actual EMS response interval. This created empirical constants that could be used to derive the response interval as had been done in previous studies [5,6]. These four time components were then added together to create a model that could estimate total EMS pre-hospital time in the absence of actual trip data. The differences between the actual EMS trip times and times derived from the updated model were mapped to gain an understanding of any spatial patterns in the underestimation and overestimation of the updated model over the study area.

## Results

### Study sample

The study sample originally contained 31,385 urgent adult patient trip records over the one year study period. After removing records with missing or invalid scene coordinates (362) and missing or invalid time stamp information (729) there were 30,294 records (96.5**%**) available for the validation study. Of these, 29,765 EMS scene locations were inside the city while 529 scene locations were outside of the city limits. The population density inside the city was roughly 3950 people per square kilometre, while outside the city it was 350 people per square kilometre.

### Objective 1: Validation of EMS modeling assumptions

**Figure 1:**
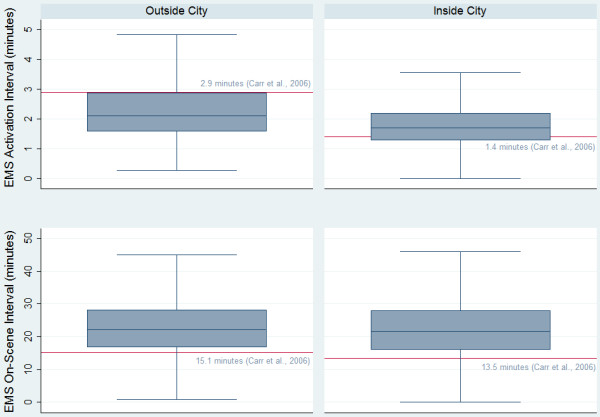
**Comparison of literature derived and actual activation and on-scene intervals.** This figure shows the actual EMS activation interval times (top two boxplots) and on-scene interval times (bottom two boxplots) in comparison to the previously published average times found through a US meta-analysis conducted by Carr et al. (2006) shown as the horizontal red line.

Figure 1 compares the actual EMS activation and on-scene intervals with the averages reported from a US meta-analysis [4]. We found that the actual median EMS activation times were lower than those previously reported for rural areas (which we consider to be outside the city) but higher for those patient trips within the city. The actual median on-scene intervals were longer than the average reported in the US by 7–8 minutes. 

**Figure 2:**
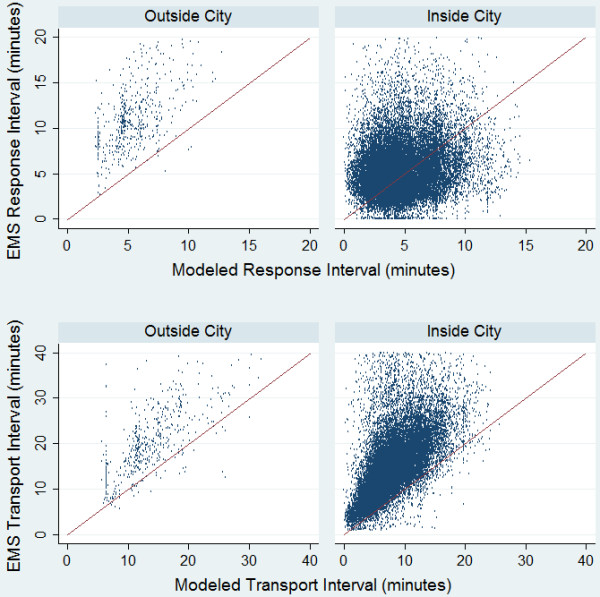
**Comparison of modeled and actual response and transport intervals.** This figure shows the actual EMS response interval times in comparison to the modeled response times (top two scatterplots) and the actual EMS transport interval times in comparison to the modeled transport times (bottom two scatterplots). The red line represents the line of agreement. If EMS times are greater than the modeled times, they fall above the line of agreement.

The top two scatter plots shown in Figure 2 compare the actual response interval with those calculated using the GIS modeled transport interval and the empirical constants. We found that most points lie above the line of agreement (red) for outside the city, showing that the actual response interval is higher than that calculated using the GIS modeled time. Within the city, the modeled and actual response times are more evenly spread around the line of agreement. The bottom two scatterplots shown in Figure 2 compare the actual transport interval with the GIS modeled transport interval. We found that most points lie above the line of agreement (red) showing that the actual transport interval is higher than that calculated using GIS both in and outside of the city.

Table 1 provides a comparison of the four travel time intervals from actual EMS records, with time components from the US meta-analysis study by Carr *et al.*[4] and derived travel time estimates using GIS. Using the Wilcoxon signed-rank test, the total modeled pre-hospital time was found to be significantly lower than the actual EMS times (p < 0.0001) both in and outside the city. 

**Table 1:** Comparison of modeled time and assumptions with actual EMS time intervals*

**(n)**	**Inside City (29765)**		**Outside City (529)**	
	Model Time**	**Actual Time**	Model Time**	**Actual Time**
Activation	1.4	1.7 (1.3-2.2)	2.9	2.1 (1.6-2.9)
Response	4.4 (3.2-5.9)	4.9 (3.5-6.7)	5.3 (4.3-7.0)	10.9 (8.9-13.6)
On-scene	13.5	21.6 (15.9-27.9)	15.1	22.1 (16.8-28.2)
Transport	7.3 (5.3-9.9)	13.3 (9.5-18.0)	13.1 (10.8-17.5)	20.0 (15.5-26.5)
Total	26.5 (23.3-30.7)	43.3 (35.7-51.9)	36.4 (33.2-42.5)	57.6 (49.4-67.2)

* All times are in minutes expressed as Median (Interquartile Range).

** Model times are the assumptions from meta-analysis for Activation and On-scene intervals and GIS derived times for Response and Transport intervals.

**Figure 3:**
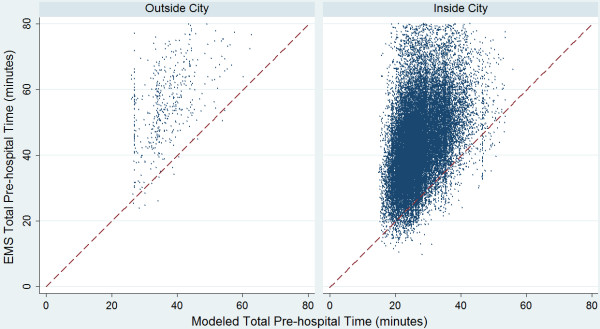
**Comparison of modeled and actual total EMS pre-hospital time.** This figure shows the actual EMS pre-hospital times in comparison to the modeled pre-hospital time. The pre-hospital time represents a total of the four time components (activation, response, on-scene and transport intervals). The dashed red line represents the line of agreement. If EMS pre-hospital times are greater than the modeled times, they fall above the line of agreement.

**Figure 4:**
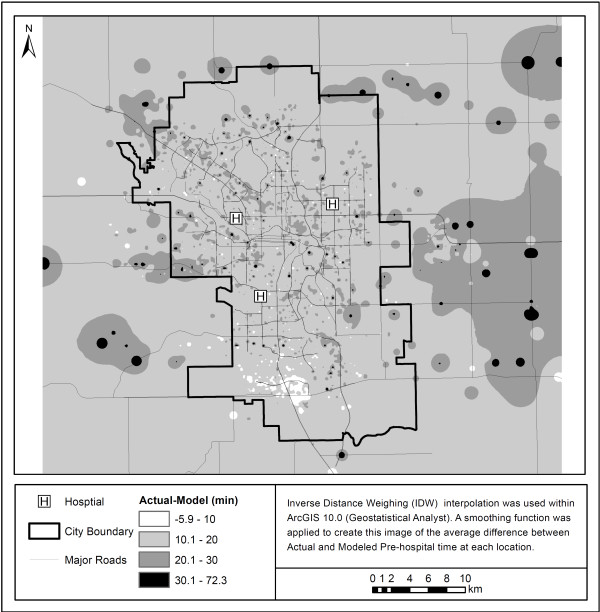
**Differences in actual vs. modeled EMS pre-hospital time using original assumptions.** This figure highlights the difference in the actual pre-hospital time and the modeled time using the original assumptions. As the colour becomes darker, the difference between the actual pre-hospital times and the modeled times becomes greater.

Figure 3 shows differences in overall model times when compared to true EMS pre-hospital times. Most points lie above the line of agreement (red) showing that the actual EMS pre-hospital time is higher than that calculated using the literature derived model both in and outside of the city. Figure 4 shows these differences in terms of geographic area. Most areas are underestimated in terms of their EMS pre-hospital times by between 10 and 20 minutes. While there are pockets of areas that are underestimated by greater than 20 minutes within the city, the majority of areas that have considerably higher than expected travel times using the model are outside of the city limits.

### Objective 2: Revising EMS modeling assumptions for a Canadian setting

Using the actual EMS records we created a revised model for the estimation of pre-hospital time using ground ambulance. We used the median activation and on-scene times from our jurisdiction in this model. Our empirical multipliers derived using linear regression to determine response time from the GIS modeled transport interval were calculated to be 0.59 (p < 0.0001, R^2^: 0.69) for areas inside of the city and 0.80 (p < 0.0001, R^2^: 0.89) for areas outside of the city. Using the findings from our validation study and equation 1) we created the following models:

3a) Urban: 1.7 minutes + (0.59*GIS transport) + 21.6 minutes + GIS transport

3b) Rural: 2.1 minutes + (0.80*GIS transport) + 22.1 minutes + GIS transport

**Figure 5:**
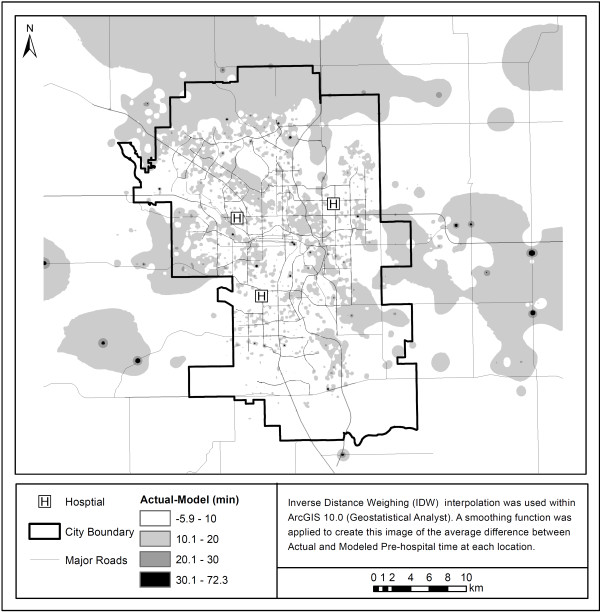
**Difference between actual vs. modeled pre-hospital time with updated EMS pre-hospital model.** This figure highlights the difference in the actual pre-hospital time and the modeled time using the revised assumptions. As the colour becomes darker, the difference between the actual pre-hospital times and the modeled times becomes greater.

When comparing Figure 4 with Figure 5 we can see that the overall difference in actual-modeled time is reduced from predominantly 10.1–20 minutes for the original model to between -6–10 minutes for the updated model. Negative differences show areas where the model was over-predicting the overall pre-hospital time. Most pre-hospital times are being under-predicted by the model. Figure 5 also shows that the updated model, although still underestimating the total pre-hospital time, more accurately represents the true pre-hospital time in most areas.

## Discussion

Our study is the first to compare widely used modeling assumptions with actual EMS trip records within a Canadian setting. A comparison of the modeling assumptions with true EMS data showed three key differences. The first was that the on-scene time in our study area was higher than that reported by the US meta-analysis. The second was that the empirical multiplier, used to derive the response time from transport time, was higher than previously reported in the US, for areas outside the immediate urban boundaries of the City of Calgary. The third was that the actual EMS pre-hospital times across our study area were significantly higher than the estimated times modeled using GIS and the original travel time assumptions. Researchers undertaking studies that evaluate the access of populations to urgent services by ground ambulance should consider that the previous assumptions may not be indicative of typical time intervals in their jurisdiction. Thus, in order to represent the pre-hospital time in our study area more accurately, we created a revised model specific to our jurisdiction that successfully reduced the overall difference between actual and modeled pre-hospital times.

The higher on-scene times we found in our study area could be applied to models across Canada. Another recent study found that the on-scene interval in Canada was similar to that noted in our study (median: 20.2 minutes; Inter Quartile Range (IQR): 14.9–27.0) [17] showing that this interval may be consistently higher than measures previously reported in the US. The debate on whether to ‘scoop and run’ or ‘stay and play’ once EMS reaches the scene has been on-going for almost two decades with mixed recommendations [18-20]. The choice of protocol used in an area will ultimately affect the scene times for a particular jurisdiction. The larger empirical constant used to derive rural response times in our study (0.89 vs. 0.4 in previous studies [5,6]) is a reflection that ambulances in areas outside the city require a greater amount of time to reach the patient scene from their originating locations. A recent study has shown that areas with low population density are at risk of delayed ambulance response times [21]. Overall, it appears that the underestimation of on-scene and transport intervals inside the city as well as an underestimation of the response intervals for areas outside the city results in the underestimation of total pre-hospital time. This underestimation of pre-hospital time is especially noticeable in areas surrounding major roadways as seen in Figures 4 and 5.

Although there have been no studies that have validated this commonly used method for evaluating pre-hospital time by ground ambulance, there have been studies that validate the GIS modeled transport interval. One study, conducted in a large Canadian city, calculated ambulance driving times using GIS for critically injured patients and found that the true transport intervals were more variable than the modeled transport interval [1]. For a single origin-destination pair they found actual transport times to be between 8 and 27 minutes, while the GIS modeled time remained constant at 13 minutes [1]. This was similar to our study findings where the range of actual travel times was greater than the single GIS transport time interval calculated for a given scene to hospital trip. This shows the importance of recognizing that all pre-hospital time estimations anchor on measures of central tendency. The value of the modeled time is that it provides a statement of expected time between scene and hospital locations, but in the real world there will always be a dispersion of values around the average. This type of estimate is useful because it provides decision makers with information regarding access to specific services. Collecting, cleaning, and analyzing historical data over large areas (e.g. across regions or countries) would be exceptionally time consuming when multiple EMS service providers are involved. Using empirical constants with GIS to model pre-hospital times can greatly facilitate a regional or national assessment of access to health care services by ground ambulance.

Evidence suggests that ambulance pre-hospital times around the world vary. The Canadian city of Montreal, Quebec reported a median response time of 8 minutes, a median on-scene interval of 16 minutes and a median transport interval of 9 minutes [22]. Monterrey, Mexico reported a median response time of 4 minutes, a median on-scene interval of 10.1 minutes and a median transport interval of 5 minutes [22]. While the city of Urmia in Iran reported on-scene intervals that were notably shorter than those reported in the US and Canada (median 5 minutes (IQR:4–7) within the city, and 7 minutes (IQR: 5–11.3) outside of the city) [23]. These pre-hospital intervals reported from different regions of the world show that a generalized model across different countries may not be appropriate. Even across the US, the pre-hospital time intervals have been shown to vary [17]. Because different jurisdictions have different geographic barriers and different EMS protocols, the travel time modeling assumptions should be adapted to the study areas under consideration.

There are limitations to creating a generalized pre-hospital travel time model. Real time traffic conditions and weather can add significantly to the response and transport interval portions of the pre-hospital time. In our study we used an entire year of data, which included EMS trips conducted at different times of day and during different seasonal conditions, to create generalized models for pre-hospital times, which incorporated these variations. When measures of access need to be considered over more specific conditions, data could be stratified by times of day, days of the week or by months of the year. Our goal in this study was to create a generalized model comparable to those used to measure national levels of population access to urgent care [8,10,12], which allows for an estimation of patient pre-hospital time in the absence of individual EMS historical data.

Based on previous methods [5] we derived a relationship between the response and transport intervals to account for the time from ambulance location to scene across the study area. The linear multipliers used in previous studies and applied here in our revised model may not be sufficiently sophisticated to capture the relationship between the response and transport intervals. The exploration of new mathematical relationships between time intervals is a potential future direction of this research. It is also important to note that there are factors that are unaccounted for that can affect this relationship. Studies have been conducted showing that higher call volumes and higher intervals of vehicles unavailable for response can increase the response time [24]. Improvements to ambulance deployment and changes in the demand volume could reduce response intervals in an area, which would require further revision to the models proposed in this study. When using generalized models across a country it is important to recognize that EMS systems in different jurisdictions have unique protocols [25-27]. The geography of an area could also affect response times. For example, areas with similar urban structures (e.g. urban sprawl) may be affected by delayed response times [28].

Finally, the reduction in the overall difference between actual and modeled pre-hospital times with our revised model is not surprising considering that data from our study area was used to create this updated model. To truly understand the effectiveness of this model we need to apply it to different jurisdictions and evaluate it in relation to actual EMS trips undertaken in these areas. In spite of these limitations we believe that the estimation of pre-hospital time using GIS is valuable for studies focused on access to urgent care, especially in the absence of actual EMS trip records. There are several messages for other jurisdictions to take away from this study. Different areas have different EMS protocols and pre-hospital benchmark goals in place that affect their activation, response, on-scene and transport intervals. It is expected that within a single country, different jurisdictions could have significantly different median pre-hospital interval times because of their unique EMS protocols. The question of which model or assumptions to use is a question of both Geography and policy.

## Conclusions

The widespread use of generalized EMS pre-hospital time assumptions based on US data may not be appropriate in a non-US context. The preference for researchers should be to use actual EMS trip records from the proposed research study area. Using this locally relevant data will create EMS pre-hospital time models that more accurately reflect the protocols within the study area. However, health services researchers often need to determine patient access across large geographic areas where EMS data is either unavailable or difficult to compile. In these cases researchers should determine which modeling assumptions more accurately reflect the EMS protocols across their study area. Our study has provided revised contemporary modeling assumptions from a large Canadian city.

## Competing interests

The authors declare that they have no competing interests.

## Authors’ contributions

ABP conceived the study, performed the analysis and drafted the manuscript. NMW participated in the design of the study, consulted on the geographic techniques to be used and provided comments on the manuscript. IEB acquired and prepared the data for analysis, provided expert EMS knowledge and commented on the manuscript. WAG and CJD participated in its design and coordination and provided comments on the manuscript. All authors read and approved the final manuscript.
